# Mortality prediction by SOFA score in ICU-patients after cardiac surgery; comparison with traditional prognostic–models

**DOI:** 10.1186/s12871-020-00975-2

**Published:** 2020-03-13

**Authors:** Abraham Schoe, Ferishta Bakhshi-Raiez, Nicolette de Keizer, Jaap T. van Dissel, Evert de Jonge

**Affiliations:** 1 Department of Intensive Care, Leiden University Medical Center, University of Leiden, Albinusdreef 2, P.O. Box 9600, 2300 RC Leiden, the Netherlands; 2 Department of Medical Informatics, Amsterdam Public Health research institute, Amsterdam Medical Center, University of Amsterdam, Amsterdam, the Netherlands; 3 National Intensive Care Evaluation (NICE) foundation, Amsterdam, the Netherlands; 4 Department of infectious diseases, Leiden University Medical Centre, University of Leiden, Leiden, the Netherlands

**Keywords:** ICU-scoring systems, ICU mortality, SOFA score, Mortality discrimination, Cardiac surgery

## Abstract

**Background:**

There are many prognostic models and scoring systems in use to predict mortality in ICU patients. The only general ICU scoring system developed and validated for patients after cardiac surgery is the APACHE-IV model. This is, however, a labor-intensive scoring system requiring a lot of data and could therefore be prone to error. The SOFA score on the other hand is a simpler system, has been widely used in ICUs and could be a good alternative.

The goal of the study was to compare the SOFA score with the APACHE-IV and other ICU prediction models.

**Methods:**

We investigated, in a large cohort of cardiac surgery patients admitted to Dutch ICUs, how well the SOFA score from the first 24 h after admission, predict hospital and ICU mortality in comparison with other recalibrated general ICU scoring systems. Measures of discrimination, accuracy, and calibration (area under the receiver operating characteristic curve (AUC), Brier score, R^2^, and Ĉ-statistic) were calculated using bootstrapping. The cohort consisted of 36,632 Patients from the Dutch National Intensive Care Evaluation (NICE) registry having had a cardiac surgery procedure for which ICU admission was necessary between January 1st, 2006 and June 31st, 2018.

**Results:**

Discrimination of the SOFA-, APACHE-IV-, APACHE-II-, SAPS-II-, MPM_24_-II - models to predict hospital mortality was good with an AUC of respectively: 0.809, 0.851, 0.830, 0.850, 0.801. Discrimination of the SOFA-, APACHE-IV-, APACHE-II-, SAPS-II-, MPM_24_-II - models to predict ICU mortality was slightly better with AUCs of respectively: 0.809, 0.906, 0.892, 0.919, 0.862. Calibration of the models was generally poor.

**Conclusion:**

Although the SOFA score had a good discriminatory power for hospital- and ICU mortality the discriminatory power of the APACHE-IV and SAPS-II was better. The SOFA score should not be preferred as mortality prediction model above traditional prognostic ICU-models.

## Background

Prediction models and scoring systems are widely used in Intensive Care medicine for prognosis, quality measures, comparison between Intensive Care Units (ICU’s) or scientific reasons. The Simplified Acute Physiology Score-II (SAPS-II) [1], Mortality Probability Model after 24 h-II (MPM_24_-II) [2], Acute Physiology and Chronic Health Evaluation–II (APACHE-II) [3] and Sequential Organ Failure Assessment (SOFA) score [4] were developed for such purposes but excluded cardiac surgery patients. Nevertheless, some of these scoring systems are also used in the cardiac surgery population admitted to the ICU [5] [6]. The only general ICU-scoring system developed to include cardiac surgery patients is the Acute Physiology and Chronic Health Evaluation–IV model (APACHE-IV), which was published in 2006 [7].

The SOFA score was initially developed as a tool to learn from the evolution of organ failure in sepsis and to assess the effects of therapies like mechanical ventilation and vasopressors on the course of organ dysfunction. It scores 1–4 points for each of the six organ systems (respiratory, circulation, renal, neurologic, hepatogenic, coagulation) [4]. The importance of the SOFA score is growing and it has been incorporated in the latest surviving sepsis campaign as a tool to describe and detect sepsis [8]. Although the SOFA score was initially not developed to predict mortality, several studies showed that SOFA has been used to predict morbidity and mortality and has been validated for that purpose in several ICU populations [9] [10]. It would be interesting to know if the SOFA score could predict mortality in the cardiac surgery population as well.

The SOFA score is much simpler compared to general ICU prediction models such as the APACHE-IV model, which requires a lot of data and lays a heavy burden on precise data acquisition. If mortality prediction could be achieved with the SOFA score as accurately as with the APACHE-IV model, use of the SOFA score would be preferable for that purpose.

The aim of the current study is to investigate, in a large retrospective cohort derived from the Dutch National Intensive Care Evaluation (NICE) registry [11] [12], how well the SOFA score on day one predicts ICU and hospital mortality in comparison to the general ICU mortality prediction models, i.e. SAPS-II, MPM_24_-II, APACHE-II, and APACHE-IV. Secondly, we wanted to investigate the contribution of the different components of the SOFA to its predictive value.

## Methods

### Data

The NICE registry collects demographic, physiological, clinical and organizational data from all 84 Dutch ICUs [12]. To ensure that the data are of a high quality, ICU employees are trained how to score patients, the data are checked before being included into the database, and data quality audits are carried out [11, 13].

We used data from cardiac surgery centers in the NICE SOFA database with an APACHE-IV admission diagnosis related to open heart surgery (see E-Supplement 2) between January 1st, 2007 and June 31st, 2018. Patients were included if they were 18 years or older and all of the following scoring systems were available: SOFA score on day one and its six individual organ scores, APACHE-IV, APACHE-II, MPM_24_-II, and SAPS-II. All readmissions within the same hospital admission were excluded from analyses.

### Severity of illness scores

Demographic data as well as all data needed to calculate the scoring systems were collected in the hospital in which the patient was admitted and were securely uploaded to the NICE registry [12]. All scoring systems were calculated according to the standards in the international literature [1] [2] [3] [4] [7]. A brief summary of the different scoring system is included in E-Supplement 1 and E-Supplement 4. We used only the SOFA score on day one because the general ICU prediction models included only data collected from the first 24 h of admission. To account for organ replacement devices that were not in common use at the time the SOFA score was developed, minor adaptations were made to the original SOFA score [4]. Consequently, we gave the maximum number of points for the renal category if the patient received continuous renal replacement therapy (CRRT) or other forms of renal replacement therapy. We gave the maximum number of points for the cardiovascular category if the patient had a left ventricular- or right ventricular assist device, an intra-aortic balloon pump (IABP) or was on veno-arterial extra corporeal membrane oxygenation (VA-ECMO). We gave the maximum number of points for the respiratory category if the patient was on veno-venous extra corporeal membrane oxygenation (VV-ECMO) or had special forms of ventilation (Nitric Oxygen (NO)-ventilation, Differential lung ventilation, Partial liquid ventilation but not prone position ventilation).

### Statistical analyses

Categorical variables are presented as percentages, and continuous variables are presented as mean and SD or as median and interquartile range (IQR) depending on the data distribution. Demographics are also provided for sub-populations based on quartiles of the SOFA score. To assess differences in distribution of continuous variables between the sub-populations based on quartiles of the SOFA score, independent t-test was used when the data was distributed normally or Mann-Whitney U test when de data was distributed not normally. Normality was tested using graphical methods. All statistical analyses were performed using R version 3.6.0. A *p* value of less than 0.05 was applied as level of significance.

## Prediction models

### Hospital mortality

The SOFA score was initially developed to quantify organ dysfunction and not to predict mortality. In order to predict hospital mortality based on SOFA score and its sub-scores, we used logistic regression modelling. To keep these models as simple as possible but also to give it a fair chance to achieve a good prognostic performance compared to the general ICU prediction models, gender and age were added to the model as covariates.

The general ICU prediction models, i.e. APACHE-IV, APACHE-II, MPM_24_-II, and SAPS-II, are logistic regression models that use different predictor variables to predict hospital mortality. These models are not stable over time [14]. To make the mortality predictions comparable to the newly defined mortality prediction models based on SOFA score, the original models were calibrated using first-level customization [14]. To this end, for each model, a logistic regression model was fitted with observed in-hospital death as the dependent variable and the logit-transformed original predictions as the independent variable.

### ICU mortality

In order to predict ICU mortality based on SOFA score and its sub-scores, we again used logistic regression modelling. Gender and age were added to the models as covariates.

The general ICU prediction models are developed to predict hospital mortality. To predict ICU mortality, logistic regression modelling was used with observed ICU mortality as the dependent variable and the logit-transformed predictions based on the original model as the independent variable.

### Performance assessment of the models

The area under the receiver operating characteristic curve (AUC) was used to describe the discrimination of the models [15]. An AUC of 0.5 indicates that the model has no discriminative power and an AUC of 1.0 indicates perfect discriminative power [15]. To compare the calibration of the models, the Hosmer-Lemeshow Ĉ-statistic was used [16]. The Hosmer-Lemeshow Ĉ-statistic assesses whether or not the observed mortality rates match the expected mortality rates in the sub-populations of the total model population [16]. The Ĉ-statistic is a χ^2^ statistic in which a *p* value of > 0.05 is considered good calibration, i.e. the difference between predicted and actual outcomes in de subgroups is low and not significantly different [16].

The Brier score was used to assess the overall accuracy of the models [17]. The Brier score is the mean squared difference between the observed and predicted outcome, which includes both discrimination and calibration aspects. The smaller the difference between observed and predicted mortalities, the lower the score, the better the model.

The performance of the models was assessed using the ordinary bootstrap method with a sample of 500 bootstraps [18]. In each sample, the performance measures were calculated and exported to a separate table. For each model, the median and 95% confidence intervals for each performance measure was defined using the 2.5th, 50th and 97.5th percentiles of the bootstrap distribution. A difference in performance measure between the models was considered statistically significant in case the median was different and the related confidence intervals did not overlap. First-level customization does not change the influence of individual covariates included in the model but calibrates their joint influence on the observed mortality [14]. Note that therefore, for the APACHE-IV, APACHE-II, MPM_24_-II, and SAPS-II models the AUC for each bootstrap sample should be the same because the order of the probabilities will not change, only the absolute magnitude of the probabilities will differ.

### Ethics

Data are encrypted such that all patient-identifying information are untraceable. The need for ethical committee approval was waived by the Central Committee on Research Involving Human Subjects, because the study was purely retrospective and used de-identified patient data (reference number W17_297 # 17.349; Medical Ethics Review Committee of the Academic Medical Center, University of Amsterdam).

## Results

**Fig. 1:**
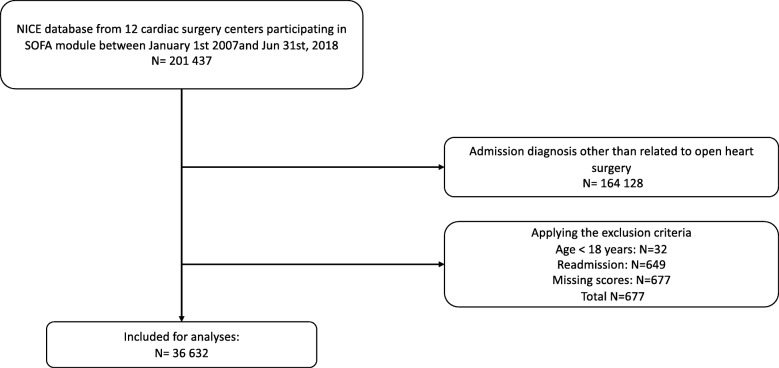
Flow chart of patient inclusion

We included 36,632 cardiac surgery patients from 12 cardiac surgery centers participating in the NICE SOFA module of whom 70.7% were men. Figure 1 shows a flowchart of the data inclusion process. Mean age was 66.8 years, 1.3% died during their ICU admission and 2.2% died in hospital. In Table 1 baseline characteristics, procedures and outcome are described, categorized by quartiles of the SOFA score (Table 1). It was not possible to distribute the number of patients evenly over the different quartiles because the data was skewed. The incidence of ICU mortality and hospital mortality is highest in the quartile with the highest SOFA scores. In these patients more emergency surgery and complex surgery is prevalent compared to the other quartiles, while the number CABG’s is lower. All patient characteristics showed unequal distribution among the sub-populations based on quartiles of SOFA score (*P* <  0.001).

**Table 1:** Demographics for all patients and stratified according to quartiles of the SOFA score

Demographics, Procedures, models & outcome	All patients	Q1 [SOFA 0–4]	Q2 [SOFA 5–6]	Q3 [SOFA 7–8]	Q4 [SOFA 9–22]
N	36,632	13,039	5486	11,848	6259
Age (mean; sd)	66.6 (11.4)	65.6 (11.9)	65.9 (11.1)	67.3 (10.7)	68.2 (11.3)
Male (%)	70.7	70.3	71	71	71
BMI (mean; sd)	27.2 (4.5)	27.2 (4.6)	27.4 (4.5)	27.1 (4.3)	26.8 (4.5)
Renal Insufficiency (%)	3.2	1.8	2	2.5	8.7
Emergency Surgery (%)	5.3	4.3	4.9	4.8	8.8
CABG (%)	51.6	56.1	58.3	51.1	37.3
Valve surgery Only (%)	24.9	24.5	22.7	25.3	26.9
Valve surgery and CABG (%)	11.9	7.6	9.2	14.1	18.9
Aorta surgery Only (%)	4.4	4.9	3.9	3.7	5.2
Myocardial surgery only (%)	1.4	1.8	0.9	1.2	1.5
Combination surgery (%)	5.8	5.2	5	4.6	10.3
Apache IV predicted mortality (%)	3	1.6	2.0	2.6	7.9
Apache III score^a^ (mean; sd)	47 (16.9)	41 (13.4)	44.8 (13.6)	47.6 (14.6)	60.4 (21.8)
Apache II predicted mortality (%)	7.6	5.5	6.6	7.6	13
Apache II score (mean; sd)	14 (4.6)	12.2 (3.7)	13.5 (3.8)	14.5 (4.0)	17.3 (5.8)
SAPS II predicted mortality (%))	12.8	8.5	10.8	12.9	23.1
SAPS II score (mean; sd)	29.4 (9.3)	25.7 (7.0)	28.2 (7.5)	30.1 (7.9)	36.7 (12.5)
MPM_24_-II predicted mortality (%)	13.5 (8.9)	11.4 (7.5)	11.4 (7.1)	14.2 (7.4)	18.7 (12.5)
ICU mortality (%)	1.3	0.1	0.2	1	5.4
Hospital mortality (%)	2.2	0.6	0.8	1.8	7.6

All patient characteristics showed unequal distribution among the subgroups based on SOFA quartiles (*P* <  0.001). ^a^ The APACHE III score is a part of the APACHE IV model

### Performance assessment of the models

Tables 2 and 3 describe the performance of the models for predicting hospital mortality and ICU mortality respectively. Measured by the AUC, the SOFA model on day one had a significantly lower discriminative power for hospital mortality compared to the APACHE-IV, APACHE-II and SAPS-II models. Also, the discriminative power of the SOFA model for ICU mortality was worse than that of the APACHE-IV, APACHE-II and SAPS-II models. The MPM_24_-II model had a significantly worse discriminative power compared to the SOFA model for both hospital mortality and ICU mortality.

**Table 2:** Performance of the models for predicting hospital mortality; *N* = 36,632 patients

Models	AUC (CI 95%)*	Brier score (CI 95%)	Ĉ-statistic (CI)*	Ĉ-statistic *p*-value
APACHE IV – model	0.851 (0.851–0.851)	0.019 (0.019–0.019)	27.0 (24.1–36.4)	< 0.0001
APACHE II – model	0.830 (0.830–0.830)	0.020 (0.19–0.20)	16.3 (12.6–24.8)	0.0308
SOFA - model	0.809 (0.808–0.810)	0.020 (0.019–0.20)	43.7 (31.5–61.1)	< 0.0001
SAPS-II - model	0.850 (0.850–0.850)	0.019 (0.019–0.019)	19.4 (11.0–33.5)	0.009
MPM_24_-II - model	0.801 (0.801–0.801)	0.020 (0.20–0.020)	30.3 (28.7–37.6)	< 0.0001

*AUC: area under the receiver operating characteristic curve; Ĉ-statistic: Hosmer and Lemeshow goodness-of-fit Ĉ-statistic; CI: 95% confidence interval

**Table 3:** Performance of the models for predicting ICU mortality; *N* = 36,632 patients

Models	AUC (CI 95%) *	Brier score (CI 95%)	Ĉ-statistic (CI)*	Ĉ-statistic *p*-value
APACHE IV – model	0.906 (0.904–0.906)	0.011 (0.011–0.011)	38.5 (30.2–54.8)	< 0.001
APACHE II – model	0.892 (0.891–0.893)	0.011 (0.011–0.011)	27.1 (14.1–35.4)	0.001
SOFA - model	0.865 (0.864–0.866)	0.012 (0.012–0.012)	16.4 (12.9–25.1)	0.030
SAPS-II - model	0.919 (0.917–0.919)	0.011 (0.011–0.012)	9.7 (5.7–21.3)	0.215
MPM_24_-II - model	0.862 (0.860–0.863)	0.012 (0.012–0.012)	7.2 (2.8–15.3)	0.462

*AUC: area under the receiver operating characteristic curve; Ĉ-statistic: Hosmer and Lemeshow goodness-of-fit Ĉ-statistic; CI: 95% confidence interval

Based on the Hosmer and Lemeshow goodness-of-fit Ĉ-statistic and related confidence intervals, the SOFA model had comparable calibration with the APACHE IV, SAPS II and MPM_24_-II models for predicting hospital mortality. APACHE II model had a significantly better calibration compared to the SOFA model (i.e. Ĉ-statistic 16.3 (12.6–24.8) versus 43.7 (31.5–61.1)). As for ICU mortality, the SOFA model showed significantly better calibration compared to the APACHE IV model (i.e. 16.4 (12.9–25.1) versus 38.5 (30.2–54.8)).

Overall, the models showed good accuracy according to the Brier score [18] [18]. The accuracy was comparable between the models for both hospital mortality (Brier score ranging between 0.019 and 0.020) and for ICU mortality (Brier score ranging between 0.011 and 0.012).

Performance measures were also calculated for the prediction models based on the six individual organ components of the SOFA model for both hospital and ICU mortality (Tables 4 and 5). For all performance measures, the overall SOFA model performed significantly better than the individual organ component models. There was no significant difference between the calibration and accuracy of the models based on individual SOFA components, however discriminative power did differ. The renal component had a significantly better discrimination compared to all other components (Renal AUC 0.771 (0.763–0.777) for ICU mortality and 0.741 (0.736–0.745) for hospital mortality). The respiratory component had a significantly poor discrimination compared to all other components.

**Table 4:** Performance of the SOFA score and its components in predicting hospital mortality; *N* = 36632patients

SOFA components	AUC (CI 95%)*	Brier score (CI 95%)	Ĉ-statistic (CI)*	Ĉ-statistic p-value
SOFA – Total	0.809 (0.808–0.810)	0.020 (0.019–0.20)	43.7 (31.5–61.1)	< 0.001
SOFA – Respiratory	0.654 (0.651–0.656)	0.022 (0.022–0.022)	10.7 (5.1–22.0)	0.170
SOFA – Coagulation	0.707 (0.702–0.709)	0.021 (0.021–0.021)	8.8 (4.0–21.1)	0.283
SOFA – Hepatogenic	0.706 (0.704–0.707)	0.021 (0.021–0.021)	9.5 (4.8–19.6)	0.267
SOFA – Circulation	0.718 (0.715–0.719)	0.021 (0.021–0.021)	16.3 (7.16–33.7)	0.021
SOFA – Renal	0.741 (0.736–0.745)	0.021 (0.021–0.021)	31.0 (19.0–44.9)	< 0.001
SOFA – Neurology	0.691 (0.689–0.692)	0.021 (0.021–0.021)	10.1 (4.3–19.3)	0.245

*AUC: area under the receiver operating characteristic curve; Ĉ-statistic: Hosmer and Lemeshow goodness-of-fit Ĉ-statistic; CI: 95% confidence interval

**Table 5:** Performance of the SOFA score and its components in predicting ICU mortality; N = 36632patients

SOFA components	AUC (CI 95%)*	Brier score (CI 95%)	Ĉ-statistic (CI)*	Ĉ-statistic p-value
SOFA – Total	0.865 (0.864–0.866)	0.012 (0.012–0.012)	16.4 (12.9–25.1)	0.030
SOFA – Respiratory	0.634 (0.630–0.637)	0.013 (0.013–0.013)	9.7 (3.1–19.9)	0.253
SOFA – Coagulation	0.728 (0.726–0.730)	0.013 (0.013–0.013)	37.6 (13.1–61.4)	< 0.001
SOFA – Hepatogenic	0.721 (0.719–0.722)	0.013 (0.013–0.013)	14.2 (8.1–23.7)	0.066
SOFA – Circulation	0.733 (0.730–0.734)	0.013 (0.013–0.013)	81.3 (33.4–134.8)	< 0.001
SOFA – Renal	0.771 (0.763–0.777)	0.013 (0.013–0.013)	40.0 (27.5–54.1)	< 0.011
SOFA – Neurology	0.668 (0.663–0.671)	0.013 (0.013–0.013)	18.7 (8.7–32.9)	0.014

*AUC: area under the receiver operating characteristic curve; Ĉ-statistic: Hosmer and Lemeshow goodness-of-fit Ĉ-statistic; CI: 95% confidence interval

## Discussion

Our main finding is that the SOFA score used as a prediction model underperforms in predicting ICU- and hospital mortality among cardiac surgery patients compared to the APACHE-IV, APACHE-II and SAPS-II models. Calibration of all models was poor for the outcome hospital mortality. From the recalibration curves (E-Supplement 3) it is clear that most models perform badly in patients with high risk, which influences the Hosmer-Lemeshow Ĉ-statistic [19]. Only the SAPS-II model and the MPM_24_-II model had good calibration for the outcome measure ICU mortality.

This study is not the first study investigating ICU prediction models in cardiac surgery patients, but it is the first study comparing these different models in a cohort of more than 36.000 patients.

Doerr et al. [5] have shown in a previous study in 2801 patients that the SOFA score and the SAPS-II had a good discriminative power for hospital mortality with an AUC of 0.85 (CI 95%; 0.81–0.88) for the SOFA score and 0.83 (0.79–0.86) for the SAPS-II model, which is different compared to our findings. Pätilä et al. [20] studied the SOFA score in 857 patients and found that the maximum SOFA score on day one predicted 30-day mortality with an AUC of 0.78 (CI 95%; 0.64–0.92) which was comparable with our finding but with a broader confidence interval, which can be explained by the low number of cases. Ceriani et al. tested the SOFA score for mortality prediction in 218 cardiac surgery patients who stayed in the ICU for > 96 h [21]. The AUC for the prediction of hospital mortality of the SOFA score on day 1 was 0.71 (CI 95%; ± 0.08).

We scored the SOFA score a little different than in the original article [4] because we included items such as (CRRT) and patients on (ECMO) giving them the maximum score possible within the respective SOFA component. It could be that other study groups treated the SOFA score differently in these patients leading to some discrepancy. We believe that the discrepancy cannot be large because it is unlikely that many patients started on day one with CRRT or ECMO. Giving patients on CRRT or ECMO the highest score within the respective SOFA component is, in our view, logical because these patients have the most severe deterioration of organ function.

From our data it is clear that most patients who died are found in the group with a SOFA score in the highest quartile. It is notable that in the last quartile surgery is of a more complex nature and has a more emergent character, while the percentage of CABG was lower, explaining the rise in mortality in this group of patients.

From the SOFA components, the renal component had the highest discriminative power followed by the circulation component. From these data we can conclude that renal insufficiency is an important determinant of mortality in cardiac surgery patients. Ceriani et al. also tested the importance of the SOFA components on day 1 and found that the cardiac component predicted mortality the best, followed by the neurologic-component and liver-component [21]. Their findings may have differed from ours because they only included patients who were admitted for more than 96 h while the median length of stay in our population was 1.8 days.

It is surprising that the SAPS-II model performed similar to the APACHE-IV model in predicting hospital mortality and was even better in predicting ICU mortality. SAPS-II does not include specific cardiac-surgical diagnostic categories and is generated from much less variables than APACHE-IV. In fact, the original SAPS-II model excluded cardiac surgery patients. The same observation has been made by Brinkman et al. [22] in the complete ICU population (i.e. all general, surgical and thoracic surgery patients).

Our data does not support the use of the SOFA score as a mortality prediction model in cardiac surgery patients. Nevertheless, we think that the SOFA score is still a valuable tool in other settings such as in the detection of sepsis [8] and the evolution of the condition of the patient [10] [4].

## Conclusion

The SOFA score has important potential advantages when compared with the APACHE-IV model being simpler and less labor intensive. However, we must conclude that in this large cohort of cardiac surgery patients the SOFA score used as a mortality prediction model underperformed compared to the APACHE-IV and SAPS-II model in predicting hospital- and ICU mortality.

## Supplementary information

**Additional file 1: E-Supplement 1.** Table with different items scored per ICU score.

**Additional file 2: E-Supplement 2.** Table with APACHE-IV diagnoses used in this study.

**Additional file 3: E-Supplement 3.** Calibration graphs of different Models with different outcomes.

**Additional file 4: E-Supplement 4.** Background information on scores and models used in this study.

## Data Availability

The data that support the findings of this study are available from the NICE registry, but restrictions apply to the availability of these data, which were used under license for the current study, and so are not publicly available. Upon reasonable request and with permission of the NICE registry, the data are possibly available from the authors.

## Abbreviations

APACHE-II: Acute Physiology and Chronic Health Evaluation–II
APACHE-IV: Acute Physiology and Chronic Health Evaluation–IV
AUC: Area under the receiver operating characteristic curve
CABG: Coronary artery bypass grafting
CRRT: Chronic renal replacement therapy
ECMO: Extra corporeal membrane oxygenation
IABP: Intra-aortic balloon pump
ICU: Intensive Care Unit
IQR: Interquartile range
MPM_24_-II: Mortality Probability Model after 24 h-II
NICE: National Intensive Care Evaluation
NO: Nitric Oxygen
SAPS-II: The Simplified Acute Physiology Score-II
SOFA: Sequential Organ Failure Assessment
VA: Veno-arterial
VV: Veno-venous
