# Supplementary material for: Mortality prediction by SOFA score in ICU-patients after cardiac surgery; comparison with traditional prognostic–models
Source: BMC Anesthesiol. 2020 Mar 13;20:65. doi: 10.1186/s12871-020-00975-2 (PMC7068937; doi:10.1186/s12871-020-00975-2)
Supplement: Supplementary file 1 — Additional file 1: E-Supplement 1. Table with different items scored per ICU score. [file 12871_2020_975_MOESM1_ESM.docx]

E-Supplement 1.

Table with different items scored per ICU score.

| Item/score | APACHE-IV | APACHE-II | MPM_24_-II | SAPS-II | SOFA |
| --- | --- | --- | --- | --- | --- |
| age | X | X | X | X |  |
| temperature | X | X |  | X |  |
| Mean arterial pressure | X | X |  |  |  |
| Systolic blood pressure |  |  |  | X |  |
| Blood pressure status  MAP combined with  vasopressors |  |  |  |  | X |
| Use of vasopressors |  |  | X |  |  |
| Heart rate | X | X |  | X |  |
| Respiratory rate | X | X |  |  |  |
| Mechanical ventilation | X |  | X | X |  |
| FiO_2_ | X | X |  |  |  |
| pO_2_ | X | X | X |  |  |
| pO_2_/FiO_2_ ratio  combined with or without mechanical ventilation |  |  |  |  | X |
| pCO_2_ | X |  |  |  |  |
| Arterial pH | X | X |  |  |  |
| Na^+^ | X | X |  | X |  |
| Potassium |  | X |  | X |  |
| Bicarbonate |  |  |  | X |  |
| Urine output | X |  | X | X |  |
| Creatinine | X | X | X |  | X |
| Urea | X |  |  | X |  |
| Blood sugar level | X |  |  |  |  |
| Bilirubin | X |  |  |  | X |
| Hematocrit | X | X |  |  |  |
| White blood cell count | X | X |  |  |  |
| Total leucocyte count |  |  |  | X |  |
| Platelets |  |  |  |  | X |
| Glascow coma score | X | X |  |  | X |
| GCS 3-5 |  |  | X |  |  |
| Albumin | X |  |  |  |  |
| Prothrombin time |  |  | X |  |  |
| Chronic diseases: |  |  |  |  |  |
| Chronic renal failure | X | X |  |  |  |
| Cirrhosis | X | X | X |  |  |
| Hepatic failure | X | X |  |  |  |
| COPD |  | X |  |  |  |
| Cardiovascular |  | X |  |  |  |
| Metastatic carcinoma | X |  | X | X |  |
| Lymphoma | X |  |  |  |  |
| Leukemia/Myeloma | X |  |  | X |  |
| Immunosupression | X | X |  |  |  |
| AIDS | X |  |  | X |  |
| Admission specifics |  |  |  |  |  |
| Pre-ICU Length of stay | X |  |  |  |  |
| Origin of patient | X |  |  |  |  |
| Readmission | X |  |  |  |  |
| Medical | X | X | X | X |  |
| Emergency surgery | X | X | X | X |  |
| Surgery | X | X |  | X |  |
| Admission diagnosis | X |  |  |  |  |
| Thrombolysis | X |  |  |  |  |
| Confirmed infection |  |  | X |  |  |
| Intracranial mass effect |  |  | X |  |  |
