# Supplementary material for: Mortality prediction by SOFA score in ICU-patients after cardiac surgery; comparison with traditional prognostic–models
Source: BMC Anesthesiol. 2020 Mar 13;20:65. doi: 10.1186/s12871-020-00975-2 (PMC7068937; doi:10.1186/s12871-020-00975-2)
Supplement: Supplementary file 2 — Additional file 2: E-Supplement 2. Table with APACHE-IV diagnoses used in this study. [file 12871_2020_975_MOESM2_ESM.docx]

| APACHE-IV diagnoses  used in study |
| --- |
| Aneurysm repair, ventricular |
| Aneurysm, thoracic aortic |
| Aneurysms, repair of other (except ventricular) |
| Aortic and Mitral valve replacement |
| Aortic valve replacement (isolated) |
| Atrial Septal Defect (ASD) Repair |
| CABG alone, coronary artery bypass grafting |
| CABG alone, redo |
| CABG redo with other operation |
| CABG redo with valve repair/replacement |
| CABG with aortic valve replacement |
| CABG with double valve repair/replacement |
| CABG with mitral valve repair |
| CABG with mitral valve replacement |
| CABG with other operation |
| CABG with pulmonic or tricuspid valve repair or replacement ONLY. |
| CABG, Minimally invasive; Mid-CABG |
| Mitral valve repair |
| Mitral valve replacement |
| Pericardiectomy (total/subtotal) |
| Pulmonary valve surgery |
| Tricuspid valve surgery |
| Tumor removal, intracardiac |
| Ventricular Septal Defect (VSD) Repair |

E-Supplement 2.
