# Supplementary material for: Mortality prediction by SOFA score in ICU-patients after cardiac surgery; comparison with traditional prognostic–models
Source: BMC Anesthesiol. 2020 Mar 13;20:65. doi: 10.1186/s12871-020-00975-2 (PMC7068937; doi:10.1186/s12871-020-00975-2)

E-supplement 3.

**Callibration graphs of different Models with different outcomes.**

AP2 is APACHE II. AP4 is APACHE IV.

Left y-axis is observed outcome. x-axis is the predicted outcome. The histogram represents the number of patients corresponding with the right y-axis.

The light-gray area of the plume is 1 sd and the dark gray area is 2 sd.

Perfect Calibration would coincide with the 45^o^ line with a small area of the plume.


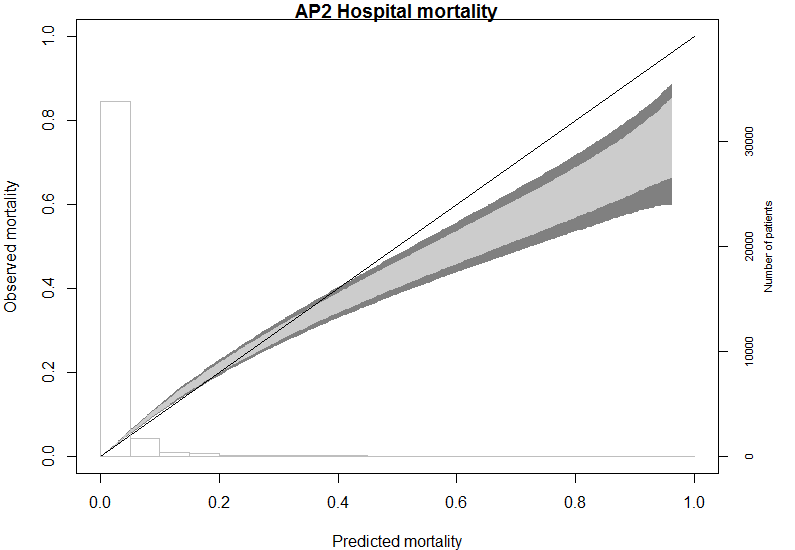

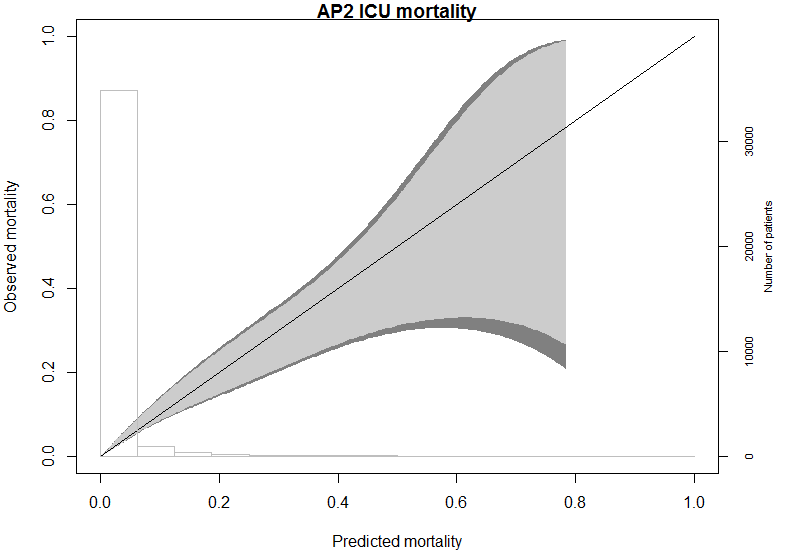

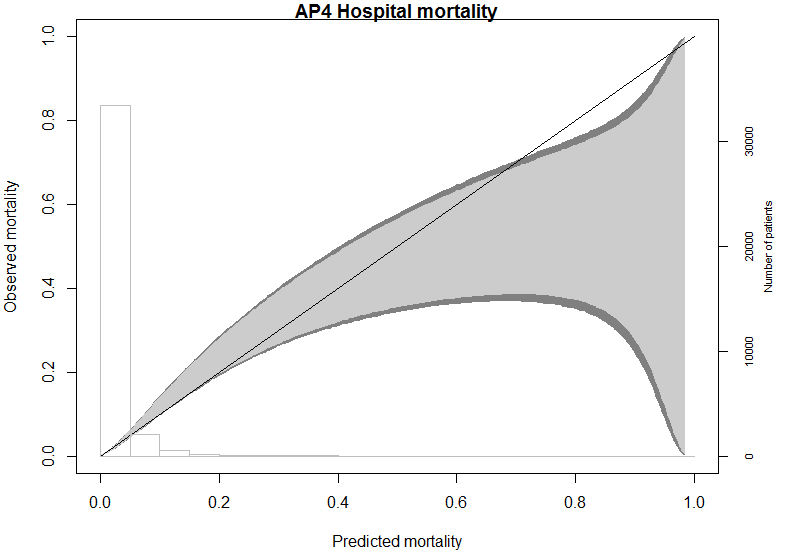

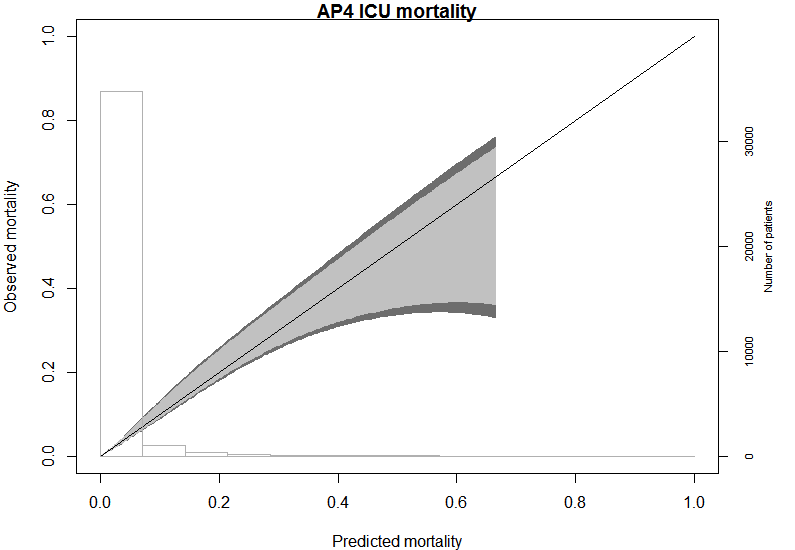

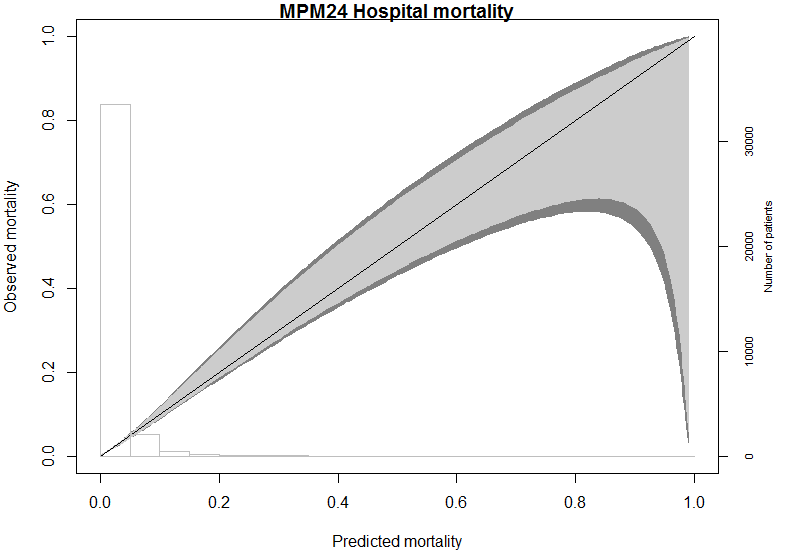

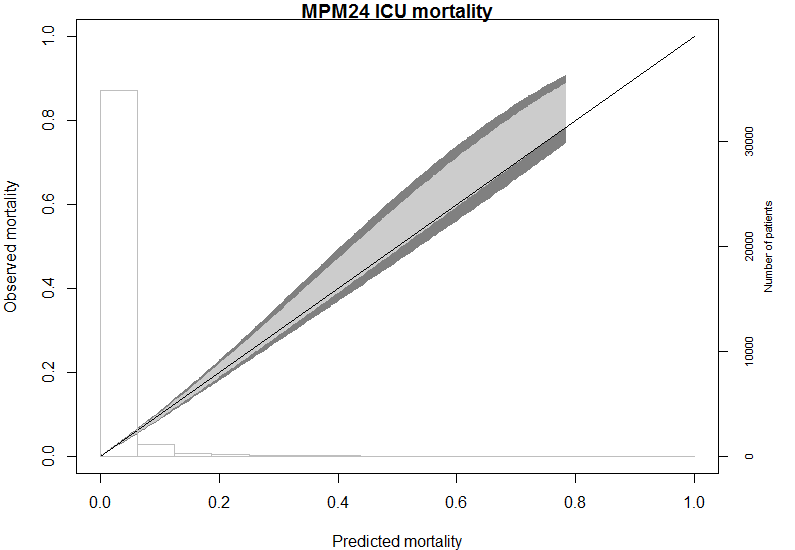


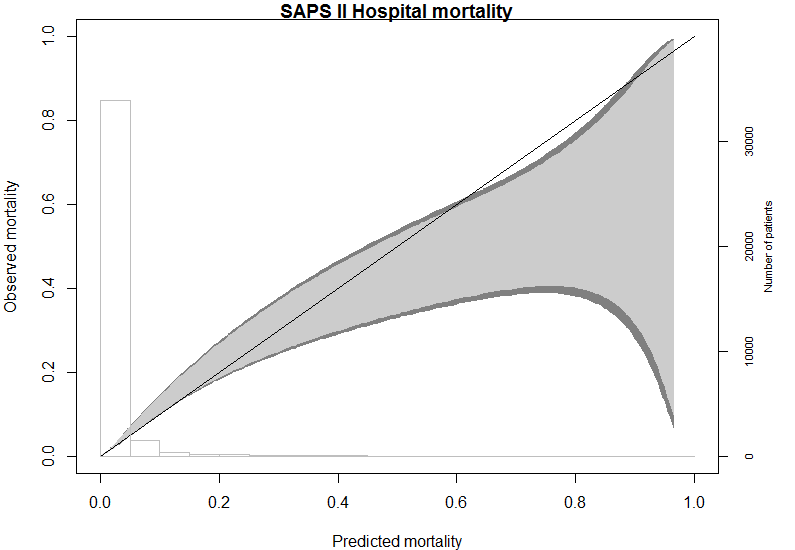


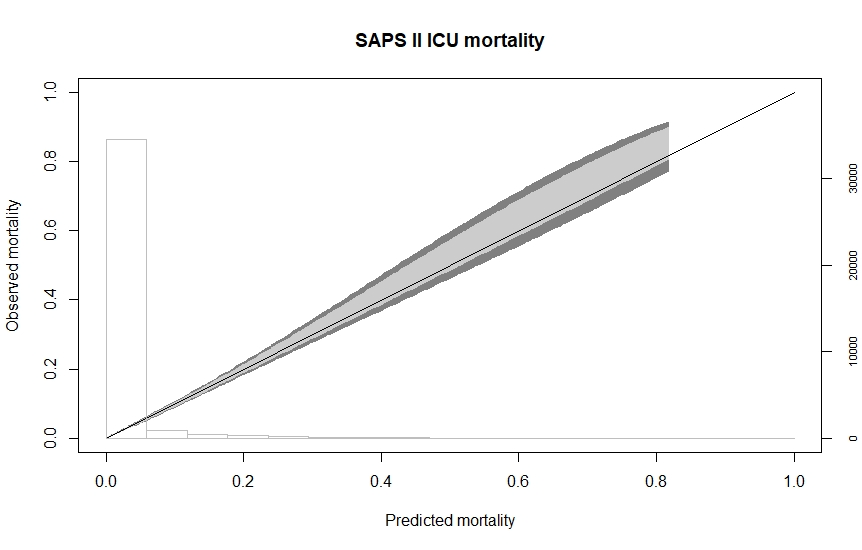


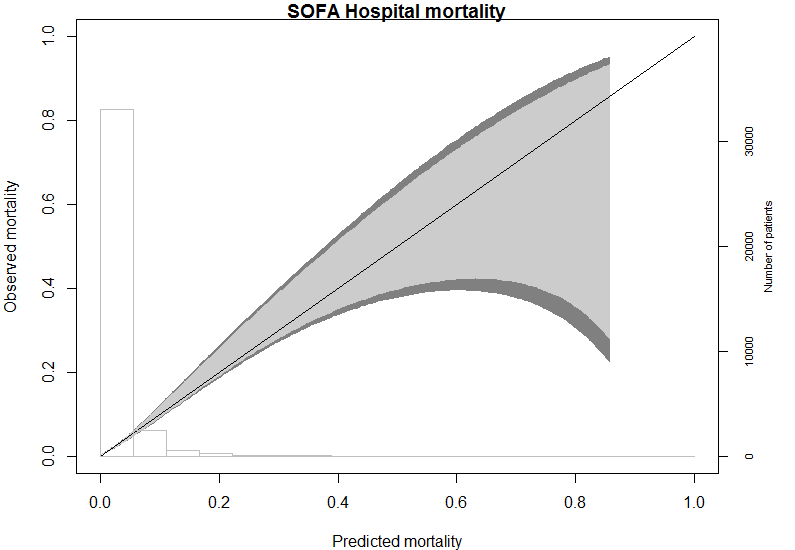
x


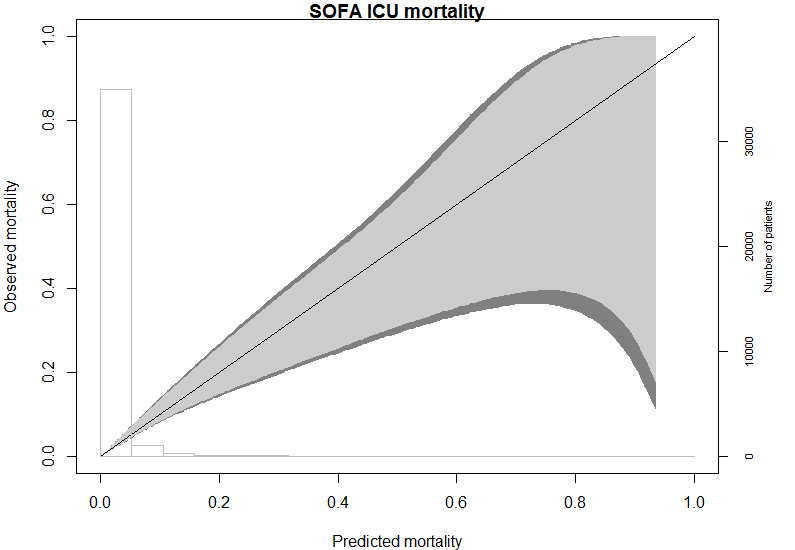

Supplement: Supplementary file 3 — Additional file 3: E-Supplement 3. Calibration graphs of different Models with different outcomes. [file 12871_2020_975_MOESM3_ESM.docx]
