# Supplementary material for: Mortality prediction by SOFA score in ICU-patients after cardiac surgery; comparison with traditional prognostic–models
Source: BMC Anesthesiol. 2020 Mar 13;20:65. doi: 10.1186/s12871-020-00975-2 (PMC7068937; doi:10.1186/s12871-020-00975-2)
Supplement: Supplementary file 4 — Additional file 4: E-Supplement 4. Background information on scores and models used in this study. [file 12871_2020_975_MOESM4_ESM.docx]

E-Supplement 4

Additional information of the Prediction Models used in the manuscript Mortality prediction by SOFA score in ICU-patients after cardiac surgery; comparison with traditional prognostic–models. (A. Schoe et al).

The text below gives a short description of the different prediction models used. Because this is not a concise review, we do encourage the reader to read the original papers and additional references to get a more complete picture. Furthermore, it is good to know that hospitals and ICU’s have to make use of third parties, such as the NICE registry, who calculate predictions, standard mortality ratios (SMR’s), keep the models up to date (recalibration etc.) or adapt the models to the local health environment where the models were not developed (see further under the heading APACHE-IV model). Although prediction models were developed initially so that individual ICU’s could predict mortality of patients in a minimum of time - almost at the bedside - the use, views and further development of prediction models necessitated the cooperation with third parties.

Because the CASUS model is not available in the NICE registry and because in is not used in Dutch ICU’s, we have not included the CASUS model in our study. We did not use the MPM_0_-II model because it only uses data collected from the moment of admission. We used the MPM_24_-II model because it uses data collected from the first 24 hours of admission like all other models used in this study. We did not use the SAPS-III score because it is not included yet in the database.

## APACHE-II model:

The APACHE-II (Acute Physiology And Chronic Health Evaluation) is a prognostic model with hospital mortality as outcome measure. It consists of three parts, an acute physiological score with 12 variables with different weights collected from the first 24 hours of ICU admission, age points and chronic health points, leading to a total score. This score, together with a distinct coefficient for every diagnostic category (like pneumonia or abdominal surgery due to perforation), and the variable emergency surgery or not, calculates an individual probability of mortality (1). The probability of death in a group can be calculated by taking the sum of the individual probabilities divided by the number of patients. From this the SMR can be calculated by dividing the actual mortality of the group by the predicted mortality of that group; a SMR larger than 1 says that the actual mortality is higher than the predicted and vice versa. The SMR can be used for comparison with other different ICU’s from other countries although recalibration needs to be done when using models in other health care environments (2). The advantage of the APACHE-II score is that it consists of relatively few variables and is relatively easy to calculate. However, for mortality prediction the admission diagnosis is needed. Which is to say that the same score can lead to different mortality prediction depending on the admission diagnosis.

## APACHE-IV model

The APACHE-IV score and model has been developed form the APACHE III model (3). It used the acute physiology score of the APACHE III model with a few variables added. The number of patients used to develop and validate the model was more than 110 000. The APACHE IV score is the total of the acute physiology score (APS – 18 variables), chronic health condition (6 variables), admission information (several variables) and admission diagnosis (116 in the original article by Zimmermann but ever expanding). The calculation of mortality and length of stay depends on the APS, the APACHE IV score and the admission diagnosis (3). The advantage of the model is that it allows comparison over a vast range of admission diagnoses including cardiac surgery. But this comes at a price. The model requires extensive high-quality data acquisition and is labor and time consuming. It also requires a third party, either commercial or non-commercial, for software development and aid in data collection. The third party is obliged to recalibrate the model, or models if they offer multiple models, for use in their own country and to update the model, which is a constant process. Even to allow decent comparison, a third party is necessary so it is clear that everyone uses the right coefficients and the data processing is not in the hands of the data owners, which could, inadvertently or not, lead to bias.

## SAPS II

The SAPS model has been developed by Le Gall et al. in 1984(4) and a second version in 1993 also by Le Gall and coworkers (5). In the first version they used 14 variables with analog weights to the APACHE system. Simplicity was their aim as the APACHE system was too complex at that time. In the second version was made up of 17 variables: 12 physiological variables, age, type of admission and three variables related to underlying disease. They included 13152 patients from 137 adult ICUs (medical and/or surgical) from 12 countries (10 European countries and 2 countries from North America) and divided these patients randomly into a developmental (65%) and validation (35%) cohort. Cardiac surgery patients were excluded. They used statistical techniques to come the weights of the different variables – LOWESS (locally weighted least series smoothing (6)) and multiple logistic regression. From this score they calculated a probability of mortality. Goodness-of-fit analysis was done using the method described by Hosmer and Lemeshow (7). The authors presented the SAPS II as a simple system for the user, estimating that it would take less than 5 minutes per patient to calculate mortality. One of the goals of the modeling process was to maintain a pure physiology-based system. However, by including three underlying chronical clinical conditions, discrimination and calibration were considerably improved. Missing values were treated as if they were within normal limits, an assumption which has been made often in model developing in the 20^th^ century for practical reasons but also an assumption which does not hold.

## MPM_0_ II and MPM_24_ II

The second version of the Mortality Prediction Model included 19 124 ICU patients (8). Cardiac surgery patients, coronary care patients and burn victims were excluded. Patients were randomly assigned to the development cohort (12 610) or the validation cohort (6514). Patients from 137 ICU’s form 12 countries (2 form North America, 10 from Europe), were included. The MPM consists of an admission model MPM_0_ and a model at 24 hours MPM_24_. The admission model contains 15 readily obtainable variables. The 24-hour model was developed on 10 357 patients still in the ICU at 24 hours, contains five of the admission variables and eight additional variables easily ascertained at 24 hours. Multiple logistic regression with backward elimination was used to derive the set of variables. Calibration was assessed using the Hosmer-Lemeshow goodness-of fit test (7). Variables whose elimination improved calibration while not significantly affecting discrimination, were considered for exclusion to further reduce the number of variables in the model. The Area under the curve for the MPM_0_ was 0.837 in the developmental set and 0.824 in the validation set. Calibration was also good with p = 0.632 in the developmental set and p = 0.327 in the validation set (a high p-value indicating good calibration).

The MPM_24_ developmental set existed of 10 357 patients, 2253 patients had either died or been discharged alive from the ICU prior to 24 hours. Model development proceeded in the same manner as for the MPM_0_ model. Calibration was good in both developmental database and validation database. Discrimination was good with an AUC-ROC of 0.844 in the development database and 0.836 in the validation database.

The MPM_0_ -II and MPM_24_ -II are included in the NICE registry.

## The SOFA-score

The SOFA-score was initially developed as a tool to learn from the evolution of organ failure in sepsis and to assess the effects of therapies like mechanical ventilation and vasopressors on the course of organ dysfunction. It scores 1-4 points for each of the six organ systems (9) (table 1.). The importance of the SOFA score is growing and it has been incorporated in the latest surviving sepsis campaign as a tool to describe and detect sepsis (10). Although the SOFA score was initially not developed to predict mortality, several studies showed that SOFA has been extensively used to predict morbidity and mortality and has been validated for that purpose in several ICU populations and among cardiac surgery patients (11) (Minne et al., 2008, #41574).

SOFA score table 1. Adapted from reference 9

| Organ  system |  | Score | | | | |
| --- | --- | --- | --- | --- | --- | --- |
|  | Variable | 0 | 1 | 2 | 3 | 4 |
| Pulmonary | Lowest Pa0_2_ (Torr)/Fi0_2_ (%) | >400 | <400 | <300 | < 200+respiratory | < 100+respiratory |
|  |  |  |  |  | support | support |
| Coagulation | Lowest platelet (1 OVmm^3^) | >150 | <150 | <100 | <50 | <20 |
| Hepatic | Highest bilirubin (jjmol/L) | <20 | 20-32 | 33-101 | 102-204 | >204 |
| Circulatory | Blood pressure status | Mean arterial | Mean arterial | Dopamine* dose <5 | Dopamine dose>5 or | Dopamine dose> 15 |
|  |  | pressure | pressure | or dobutamine any | epinephrine<0.1 or | or epinephrine>0.1 or |
|  |  | (mmHg) >70 | (mmHg) <70 | dose | norepinephrine <0.1 | norepinephrine >0.1 |
| Neurologic | GCS | 15 | 13-14 | 10-12 | 6-9 | <6 |
| Renal | Highest creatinine level (/L/mol/L) | <110 | 110-170 | 171-299 | 300-440 | >440 |
|  | Total urine output (mL/24 h) |  |  |  | <500 | <200 |
| Score | 0-6 | 7-9 | 10-12 | 13-14 | 15 | 15-24 |
| Score % | <10 | 15-20 | 15-20 | 50-60 | >80 | >90 |

PaO,: (Toit) arterial oxygen tension; FiO,: Fractional concentration of inspired oxygen; GCS: Glasgow coma score

Literature:

1. Knaus, WA, Draper, EA, Wagner, DP et al.: APACHE II: a severity of disease classification system. *Crit Care Med* 1985; 13:818-829

2. Bakhshi-Raiez, F, Peek, N, Bosman, RJ et al.: The impact of different prognostic models and their customization on institutional comparison of intensive care units. *Crit Care Med* 2007; 35:2553-2560

3. Zimmerman, JE, Kramer, AA, McNair, DS et al.: Acute Physiology and Chronic Health Evaluation (APACHE) IV: hospital mortality assessment for today’s critically ill patients. *Crit Care Med* 2006; 34:1297-1310

4. Le Gall, JR, Loirat, P, Alperovitch, A et al.: A simplified acute physiology score for ICU patients. *Crit Care Med* 1984; 12:975-977

5. Le Gall, JR, Lemeshow, S, Saulnier, F: A new Simplified Acute Physiology Score (SAPS II) based on a European/North American multicenter study. *JAMA* 1993; 270:2957-2963

6. Cleveland, WS: Robust Locally Weighted Regression and Smoothing Scatterplots. *Journal of the American Statistical Association* 1979; 74:829-836

7. Hosmer, DW, Hosmer, T, Le Cessie, S et al.: A comparison of goodness-of-fit tests for the logistic regression model. *Stat Med* 1997; 16:965-980

8. Lemeshow, S, Teres, D, Klar, J et al.: Mortality Probability Models (MPM II) based on an international cohort of intensive care unit patients. *JAMA* 1993; 270:2478-2486

9. Vincent, JL, Moreno, R, Takala, J et al.: The SOFA (Sepsis-related Organ Failure Assessment) score to describe organ dysfunction/failure. On behalf of the Working Group on Sepsis-Related Problems of the European Society of Intensive Care Medicine. *Intensive Care Med* 1996; 22:707-710

10. Singer, M, Deutschman, CS, Seymour, CW et al.: The Third International Consensus Definitions for Sepsis and Septic Shock (Sepsis-3). *JAMA* 2016; 315:801-810

11. Ceriani, R, Mazzoni, M, Bortone, F et al.: Application of the sequential organ failure assessment score to cardiac surgical patients. *Chest* 2003; 123:1229-1239
